# This Special Issue

**DOI:** 10.4103/0972-9941.19269

**Published:** 2005-10

**Authors:** Tehemton E. Udwadia

**Affiliations:** Emeritus Professor of Surgery, Grant Medical College & J.J. Hospital Head, Department of M.A.S., P. D. Hinduja National Hospital

At its very first meeting the Editorial Board of the JMAS had decided to bring out one Special Issue of the Journal every year. The Journal could not have asked for a better, more balanced, more thought provoking first Special Issue than this one on Laparoscopy in Urology compiled by our Guest Editors Professor Ashok K. Hemal and Dr Rajeev Kumar. The Journal is indebted to them for this significant contribution to M.A.S. literature.

Surgeons in every surgical speciality are making forays into “laparoscopic” surgery to try and establish to which procedures and to what extent M.A.S. can be of significant patient benefit in their speciality, be it urologic, pediatric, neuro, colo-rectal, G.I. E.N.T., thoracic, cardiovascular, endocrine, plastic, and of course the father of all surgical speciality general surgery. In a short fifteen years there has been an upheaval and new revelation in the mental and practical approach to all surgery.

The urologic surgeon was the first surgical specialist to explore and evaluate the field of minimal access surgery, well before the advent of laparoscopic surgery. Much before other surgeons awoke to the possibilities and impact of minimal access surgery, the urologist had documented and established the unquestioned benefits of trans-urethral prostate resection viz a viz open surgery, of trans-urethral surgery for bladder tumours in appropriate case, of PCNL and ESWL in urinary calculi. These decades of mental conditioning and expertise in endoscopy are reflected in the maturity and approach evident in every one of the articles which discuss established procedures, other procedures under evaluation or of possible future impact. Each article bears the stamp of thought, dedication, experience and authority.

To one in surgical practice for fifty years, all surgical specialities stem from general surgery and are interwoven and inter-related. This answers the query some may have to this Journal's Special Issue on a speciality most of its subscribers do not practice. Each one of us in our own speciality, is so focused on what we do and how we do it that we could easily get blinkered. As Gandhiji had emphasized, it helps to keep all our windows open so that the winds of other thoughts and methods could help in the cross-pollination which is the basis of all evolution and progress.

This issue transcends any M.A.S. speciality - it goes to the root of all surgery, especially M.A.S. In an age where surgery is both patient and industry driven the pit-falls of M.A.S. are numerous and potentially catastrophic. Each article emphasizes fundamentals common to every M.A.S. speciality. Insecurity, ego, lucre are poor indications for entering the field of laparoscopic surgery. Each M.A.S. procedure, whatever, has a learning curve which can only be surmounted by dedicated training and expertise, and expertise requires far more than reading journals or attending (quite often in far away lands!) four or five day “training workshops”. Every M.A.S. procedure, whatever, as it evolves, must be honestly and pragmatically evaluated in terms of safety, outcome, cost and applicability in the local environment, against what till then is the “gold standard” of open surgery. All surgery has the same fundamentals, and as we M.A.S. surgeons distance ourselves from the patient with our long instruments and even furthermore in the foreseeable future with robots, let us not forget that the patient is not an object but a fellow human, who would greatly benefit by seeing the face and holding the hand of his surgeon.

